# TRPV1 mediates cellular uptake of anandamide and thus promotes endothelial cell proliferation and network-formation

**DOI:** 10.1242/bio.20149571

**Published:** 2014-11-13

**Authors:** Nicole A. Hofmann, Sonja Barth, Markus Waldeck-Weiermair, Christiane Klec, Dirk Strunk, Roland Malli, Wolfgang F. Graier

**Affiliations:** 1 Institute for Molecular Biology and Biochemistry, Medical University of Graz, A-8010 Graz, Austria; 2 Experimental and Clinical Cell Therapy Institute, Paracelsus Medical University, A-5020 Salzburg, Austria

**Keywords:** transient receptor potential vanilloid 1, TRPV1, anandamide, AEA, endothelial colony-forming cells, ECFC, anandamide transport, proliferation, network-formation, angiogenesis

## Abstract

Anandamide (N-arachidonyl ethanolamide, AEA) is an endogenous cannabinoid that is involved in various pathological conditions, including cardiovascular diseases and tumor-angiogenesis. Herein, we tested the involvement of classical cannabinoid receptors (CBRs) and the Ca^2+^-channel transient receptor potential vanilloid 1 (TRPV1) on cellular AEA uptake and its effect on endothelial cell proliferation and network-formation. Uptake of the fluorescence-labeled anandamide (SKM4-45-1) was monitored in human endothelial colony-forming cells (ECFCs) and a human endothelial-vein cell line (EA.hy926). Involvement of the receptors during AEA translocation was determined by selective pharmacological inhibition (AM251, SR144528, CID16020046, SB366791) and molecular interference by TRPV1-selective siRNA-mediated knock-down and TRPV1 overexpression. We show that exclusively TRPV1 contributes essentially to AEA transport into endothelial cells in a Ca^2+^-independent manner. This TRPV1 function is a prerequisite for AEA-induced endothelial cell proliferation and network-formation. Our findings point to a so far unknown moonlighting function of TRPV1 as Ca^2+^-independent contributor/regulator of AEA uptake. We propose TRPV1 as representing a promising target for development of pharmacological therapies against AEA-triggered endothelial cell functions, including their stimulatory effect on tumor-angiogenesis.

## INTRODUCTION

Anandamide (AEA; N-arachidonyl ethanolamide *Sanskrit* for bliss) is the most prominent and most extensively studied endocannabinoid. AEA activates distinct G-protein coupled receptors (GPR), known as cannabinoid receptors (CBRs), including CB_1_R, CB_2_R and GPR55 as well as the Ca^2+^-channel transient receptor potential vanilloid 1 (TRPV1) causing multiple biological effects on different tissues (Howlett et al., 2002; Pertwee et al., 2010; Galve-Roperh et al., 2013). Exemplarily, AEA mediates neuronal regulation, inflammatory response (Howlett et al., 2002; Pertwee et al., 2010) and cardiovascular effects including the dilation of blood vessels, cardio protection after cardiac ischemia/infarction and tumor-angiogenesis (Deutsch et al., 1997; Wagner et al., 1997; Pisanti et al., 2011). Importantly, because these receptors have been recently found to be functionally localized intracellularly (Rozenfeld and Devi, 2008; Brailoiu et al., 2011; den Boon et al., 2012; Fowler, 2013), the cellular uptake mechanisms of AEA obviously gained importance for the physiological function of this endocannabinoid. Since essential cellular mechanisms comprising how endocannabinoids bypass the plasma membrane remain unresolved the development of pharmacological therapies is hampered (McFarland and Barker, 2004; Fowler, 2013).

Evidence for different hypothetic AEA translocation mechanisms have been reported ranging from involvement of a putative transporting protein called fatty acid amid hydrolase (FAAH) to FAAH-independent facilitated or even passive diffusion (Hillard and Campbell, 1997; Glaser et al., 2003; Fegley et al., 2004; McFarland and Barker, 2004; Fowler, 2013; Björklund et al., 2014). In these studies, a compound called AM404 was originally described to be an endogenous cannabinoid reuptake inhibitor (Costa et al., 2006). However, subsequent data have been inconclusive and rose doubts whether an AEA transporter even existed (Glaser et al., 2003; Fegley et al., 2004). Not the least these doubts arose because the AM404 effect could not uniquely be assigned to FAAH inactivation, but inhibition of cyclooxygenase (Fowler, 2013; Björklund et al., 2014) and TRPV1 Ca^2+^-channeling function (Högestätt et al., 2005).

TRPV1 is a tetramer protein each subunit composed of six transmembrane spanning domains and is known to contribute to acute and persistent pain (Caterina et al., 1997; Starowicz et al., 2007; Basbaum et al., 2009). Up to now it is assumed that AEA binds to the intracellular face of the capsaicin receptor TRPV1 leading to opening of the Ca^2+^ permeable channel pore (De Petrocellis et al., 2001; van der Stelt et al., 2005). Therefore, TRPV1 has been used as a tool to indirectly monitor intracellular AEA and its uptake based on increasing cytoplasmic Ca^2+^-levels (De Petrocellis et al., 2001; Ligresti et al., 2010). However, this notion has been recently challenged by evidence showing that TRPV1 could be activated at the outer pore by a bivalent tarantula toxin (Bohlen et al., 2010). Thrillingly, two reports published back to back have subsequently refined structural analysis of TRPV1 using electron cryo-microscopy revealing a hydrophobic binding pocket for capsaicin and AEA that is accessible from the extracellular side (Cao et al., 2013; Liao et al., 2013), thus indicating that these compounds access TRPV1 from the outside.

Based on the intracellular location of the endocannabinoid receptors (Rozenfeld and Devi, 2008; Brailoiu et al., 2011; den Boon et al., 2012; Fowler, 2013), the AEA transporter might represent a bottleneck for AEA action and, therefore, provides a promising target for the development of pharmacological therapies for various AEA-related function in the progression of diseases. It has been reported that AEA is involved in tumor-angiogenesis and can be produced in different sources of endothelial progenitor cells (EPCs) including human peripheral blood, umbilical cord and aortic derived endothelial cells (Opitz et al., 2007; Pisanti et al., 2007; Pisanti et al., 2011). The so called vessel wall-derived endothelial colony-forming cells (ECFCs) are a subtype of EPCs that have a high clonogenic and proliferation potential and show a robust vessel-forming capacity *in vivo* (Ingram et al., 2005; Yoder et al., 2007; Reinisch et al., 2009). These characteristics make ECFCs a favorable cellular tool to study the potential influence of AEA on cell behavior and yield a promising target for pro- and anti-angiogenic therapies.

In the present study a fluorescence-labeled analogue of AEA (SKM4-45-1) (Muthian et al., 2000) was used to monitor the AEA uptake into ECFCs and the immortalized human endothelial vein cell line (EA.hy926). The involvement of CB_1_R, CB_2_R, GPR55 and TRPV1 during AEA translocation was determined by using pharmacological and genetic inhibition and overexpression, respectively. We describe herein that exclusively TRPV1 but not the CBRs/GPR55 is fundamental for uptake of AEA into endothelial cells and, thus, for AEA-mediated endothelial cell proliferation and network-formation *in vitro*. These findings highlight the importance of TRPV1 for AEA uptake and its subsequent cellular function and presents TRPV1 as essential regulator of the AEA-induced pro-angiogenic effects. Therefore, our study suggests TRPV1 to be a promising therapeutic target for various AEA-related pathologies such as cardiovascular diseases and tumor-angiogenesis.

## RESULTS

### Revealing the involved receptor for AEA uptake in endothelial cells

**Fig. 1.**
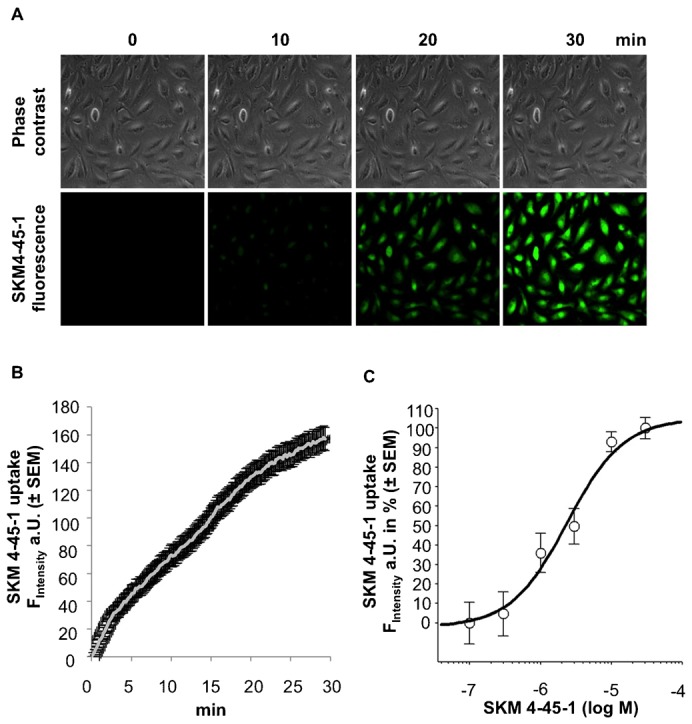
Endothelial colony-forming cells (ECFCs) accumulate the anandamide (AEA) analog SKM4-45-1 in a time and concentration dependent manner. (A) Representative images of phase contrast (upper images) and SKM4-45-1-derived fluorescence in ECFCs (green, lower images) at incubation time-points 0, 10, 20 and 30 min (at 20× magnification). (B) Kinetics of SKM-45-1 uptake in ECFCs. Increase in fluorescence was documented every 10 sec throughout 30 min. (n = 8; ± SEM). (C) Concentration response of SKM4-45-1 uptake in ECFCs. Increase in fluorescence intensity with various concentrations of SKM4-45-1 (0.1, 0.3, 1, 3, 10, 30 µM) after 30 min (± SEM) of incubation in % of the maximal fluorescence intensity is blotted (n = 3).

To allow a quantifiable visualization of the cellular uptake of AEA in real time, the cellular accumulation of SKM4-45-1, a labeled non-fluorescent analogue of AEA that is cleaved by intracellular esterases and becomes fluorescent (Muthian et al., 2000) (supplementary material Fig. S1A) was measured fluorometrically in human ECFCs and the human umbilical vein endothelial cell-derived cell line EA.hy926. ECFCs accumulate SKM4-45-1 in a time- (Fig. 1A,B) and concentration- (Fig. 1C) dependent manner with a k_d_ of 2.63 (0.24–28.47) µM. Similar data were obtained in the endothelial cell line EA.hy926 (supplementary material Fig. S1B). Hence, the proposed pan-transport inhibitor AM404 of which the exact target is unknown (Costa et al., 2006), reduced the uptake of SKM4-45-1 by 54.9 ± 0.4% (supplementary material Fig. S1C). These data suggested that AEA is actively shuttled into the cell by a specific saturable transport mechanism.

**Fig. 2.**
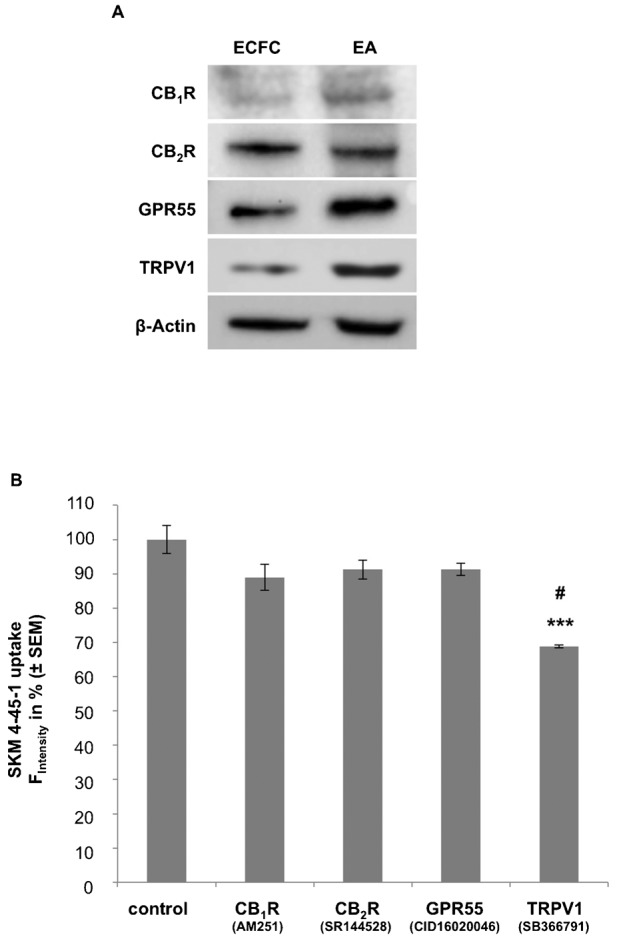
ECFCs express various (endo)cannabinoid receptors (CBR) of those the pharmacological inhibition exhibits different effects on the uptake of the AEA analog SKM4-45-1. (A) Western blots representing protein levels of CB1R, CB2R, G-protein coupled receptor 55 (GPR55), transient receptor potential vanilloid 1 (TRPV1) and β-Actin in ECFCs and EAhy.926 (EA) cells. (B) Columns represent SKM 4-45-1 (1 µM) uptake in ECFCs with vehicle or antagonists against CB1R (AM251, 0.1 µM; n = 3), CB2R (SR144528, 1 µM; n = 4), GPR55 (CID16020046, 20 µM; n = 4) and TRPV1 (SB366791, 10 µM; n = 4) after 30 minutes of incubation. Values show % fluorescence as compared to vehicle control (± SEM); *** indicates p ≤ 0.001 significance as compared to vehicle control, # indicates p ≤ 0.01 significance as compared to other treatments.

We hypothesized that the uptake of AEA could be regulated by one or more CBRs. Thus, protein levels of the known CBRs in ECFCs and EA.hy926 were detected by western blot and revealed the expression of CB_1_R, CB_2_R, GPR55 and TRPV1 in both cell types (Fig. 2A). To explore the involvement of these cannabinoid receptors during the AEA uptake, the effect of pharmacological inhibitors against CB_1_R (AM251; 0.1 µM), CB_2_R (SR144528; 1 µM), GPR55 (CID16020046; 20 µM) and TRPV1 (SB366791; 10 µM) on cellular SKM4-45-1 accumulation was tested. While inhibition of CB_1_R (11.0 ± 3.8%), CB_2_R (8.7 ± 2.8%) and GPR55 (8.7 ± 1.8%) only marginally reduced SKM4-45-1 uptake, the pharmacological blockage of TRPV1 by SB366791 strongly diminished intracellular SKM4-45-1 accumulation by 31.2 ± 0.4% (Fig. 2B). Similar findings were obtained in EA.hy926 cells (supplementary material Fig. S2A).

In order to determine an engagement of FAAH in the AEA uptake, this enzyme was selectively inhibited with methyl arachidonyl fluorophosphonate (MAFP) that did not affect SKM4-45-1 uptake in ECFCs (supplementary material Fig. S2B), thus pointing to a FAAH-independent uptake mechanism for AEA in ECFCs. Moreover, to verify whether the inhibitory effect of the TRPV1 inhibitor SB366791 on AEA uptake in ECFCs is false positive due to an unknown inhibitory effect of SB366791 on intracellular esterases, its effect on esterase-mediated cleavage of Fluo-4/AM was tested. SB366791 did not affect esterase-dependent cleavage of Fluo-4/AM in ECFCs (supplementary material Fig. S2C), thus, an inhibitory effect of SB366791 on intracellular esterases could be ruled out. These data point to TRPV1 as a key player during AEA transport in endothelial cells. Therefore, we further focused on the obvious involvement of TRPV1 during AEA uptake in endothelial cells.

### TRPV1 drives AEA uptake in endothelial cells

**Fig. 3.**
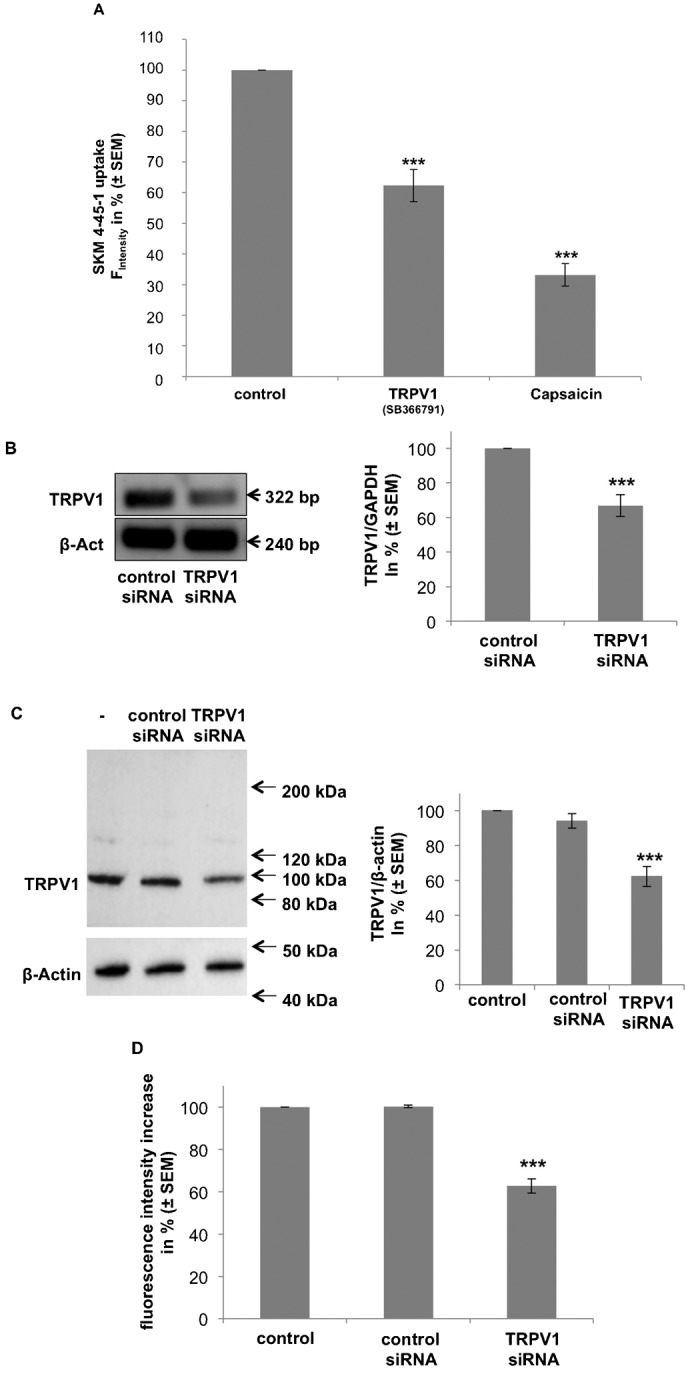
SKM4-45-1 uptake in ECFCs is diminished by either the inhibition of TRPV1, the TRPV1 agonist capsaicin, and a siRNA-mediated down-regulation of TRPV1. (A) Comparison of the inhibitory effects of the TRPV1 antagonist SB366791 and the TRPV1 agonist capsaicin. SKM4-45-1 (1 µM) was added to ECFCs treated with vehicle, antagonist (SB366791, 10 µM; n = 4) or agonist (Capsaicin, 0.1 µM; n = 3) of TRPV1. Values show % fluorescence as compared to vehicle control (± SEM); *** indicates p ≤ 0.001 significance as compared to vehicle control. (B) Genetic knock-down of TRPV1 mRNA levels. EA.hy926 cells were treated with siRNA against TRPV1. Effectiveness of knock-down was verified by RT-PCR (left panel) and quantitative real time (q) PCR (right panel). Values show % TRPV1 corrected for GAPDH as compared to nonspecific siRNA controls (n = 3; ± SEM); *** indicates p ≤ 0.001 significance as compared to siRNA control. (C) Genetic knock-down of TRPV1 protein level. EA.hy926 cells were treated with non-specific siRNA or siRNA against TRPV1. Effectiveness of knock-down was verified by western blot using antibodies against TRPV1 and β-Actin (left panel), as indicated, and quantified by ImageJ® (right panel). Values show % TRPV1 corrected for β-Actin as compared to untreated and non-specific siRNA controls (n = 3; ± SEM); *** indicates p ≤ 0.001 significance as compared to control. (D) Columns represent SKM4-45-1 uptake in untreated control cells and EA.hy926 cells treated with non-specific siRNA or siRNA against TRPV1, as indicated. SKM4-45-1 fluorescence was measured 30 minutes after incubation (n = 3; ± SEM); *** indicates p ≤ 0.001 significance as compared to control.

Next, we evaluated whether the observed TRPV1-mediated AEA uptake is genuinely affected by capsaicin binding to the recently described extracellular hydrophobic binding site (Cao et al., 2013; Liao et al., 2013). Upon simultaneous application of SKM4-45-1 (1 µM) with capsaicin (0.1 µM) the accumulation of SKM4-45-1 in ECFCs and EA.hy926 was significantly reduced by 66.8 ± 3.6% and 61.2 ± 4.6%, respectively (Fig. 3A; supplementary material Fig. S2A). To confirm the data obtained with the TRPV1 antagonist SB366791, TRPV1 expression was diminished by transient transfection with respective siRNA. Efficiency of siRNA against TRPV1 expression was depicted by quantitative real time (RT)-PCR (Fig. 3B) and western blot (Fig. 3C) and revealed a protein knock-down efficiency of 37.7 ± 5.8%. The knock-down efficiency on TRPV1 expression by specific siRNA correlated well with a reduced SKM4-45-1 uptake by 37.3 ± 3.4% (Fig. 3D).

**Fig. 4.**
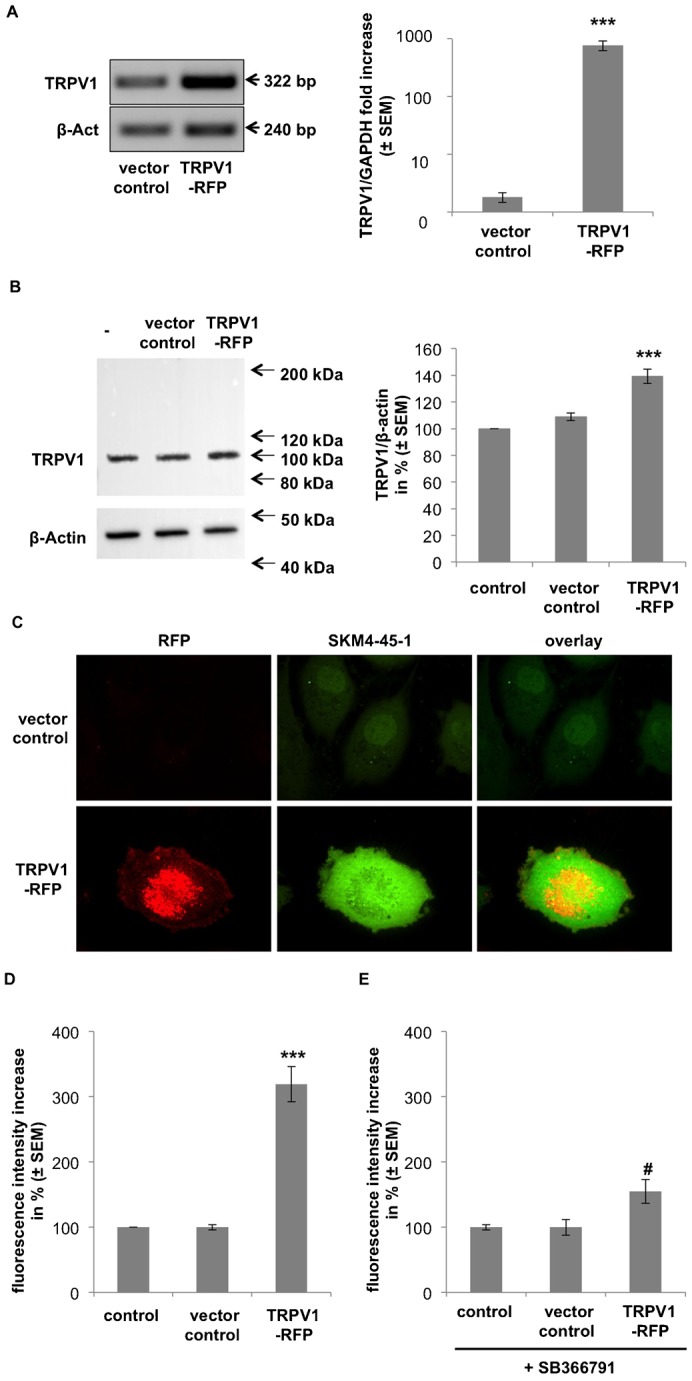
Overexpression of TRPV1 leads to an increase in SKM4-45-1 uptake in human endothelial cells. (A) Verification of TRPV1 mRNA overexpression in EA.hy926 cells. Cells were transfected with a TRPV1 encoding plasmid conjugated to a red fluorescence protein (TRPV1-RFP) or an empty vector control. Effectiveness of overexpression was verified by RT-PCR (left panel) and quantitative real time (q) PCR (right panel). Values show % TRPV1 corrected for GAPDH as compared to empty vector (vector control) (n = 3; ± SEM); *** indicates p ≤ 0.001 significance as compared to control. (B) Verification of TRPV1 protein overexpression in EA.hy926 cells. Effectiveness of TRPV1-RFP overexpression was verified by western blot using antibodies against TRPV1 and β-Actin, as indicated, and quantified by ImageJ® (right panel). Values show % TRPV1 corrected for β-Actin as compared to untreated and empty vector controls (n = 3; ± SEM); *** indicates p ≤ 0.001 significance as compared to control. (C) Single cell fluorescence images of SKM4-45-1 accumulation in control EA.hy926 cells and cells overexpressing TRPV1-RFP (at 100× magnification). RFP expression (left panel; red signal) and SKM4-45-1 uptake (middle panel; green signal) was measured after 30 minutes incubation and single channel pictures where overlaid (right panel). (D) Columns represent SKM4-45-1 accumulation in untransfected EA.hy926 cells, vector control cells and cells overexpressing TRPV1-RFP. SKM4-45-1 uptake was measured after 30 minutes incubation. Values show % fluorescence as compared to untransfected controls (n = 3; ± SEM); *** indicates p ≤ 0.001 significance as compared to untransfected control. (E) Columns represent SKM4-45-1 accumulation in control EA.hy926 cells, cells overexpressing TRPV1 in the presence of SB366791. SKM4-45-1 uptake in untransfected, empty vector control transfected and TRPV1-RFP transfected EA.hy926 was measured after 30 minutes simultaneous incubation of 1 µM SKM4-45-1 with 10 µM SB366791. Values show % fluorescence as compared to untransfected controls (n = 3; ± SEM). There is no significant difference to untransfected controls. # indicates p ≤ 0.01 significant decrease as compared to TRPV1-RFP transfected cells without SB366791 treatment.

Hence, the effect of TRPV1 over-expression was tested on SKM-4-45-1 uptake in endothelial cells transiently transfected with pcDNA3^−^ encoding for TRPV1 conjugated to a red fluorescence protein (TRPV1-RFP). Successful overexpression as compared to untreated and empty vector controls was verified by quantitative real time (RT)-PCR (Fig. 4A) and western blot (Fig. 4B). In fact, TRPV1-RFP transfected cells showed a strong increase in SKM4-45-1 uptake by 219.4 ± 26.9% as compared to empty vector controls (Fig. 4C,D). A similar effect was also observed with TRPV1 overexpression in the human cancer cell lines, HeLa and Hek293 (supplementary material Fig. S3A). However, the TRPV1 inhibitor SB366791 prevented TRPV1-RFP transfected EA.hy926 to perform a significant increase of SKM4-45-1 uptake as compared to controls (Fig. 4E). In order to determine the influence of Ca^2+^ that is carried by TRPV1 on the contribution of TRPV1 to AEA uptake, SKM4-45-1 internalization was measured in nominal Ca^2+^-free (i.e. EGTA buffered) medium. Removal of extracellular Ca^2+^ did not affect SKM4-45-1 uptake, thus, pointing to a capsaicin-sensitive but Ca^2+^-independent engagement of TRPV1 to AEA uptake (supplementary material Fig. S3B).

### TRPV1-mediated AEA uptake promotes endothelial cell proliferation and network-formation

**Fig. 5.**
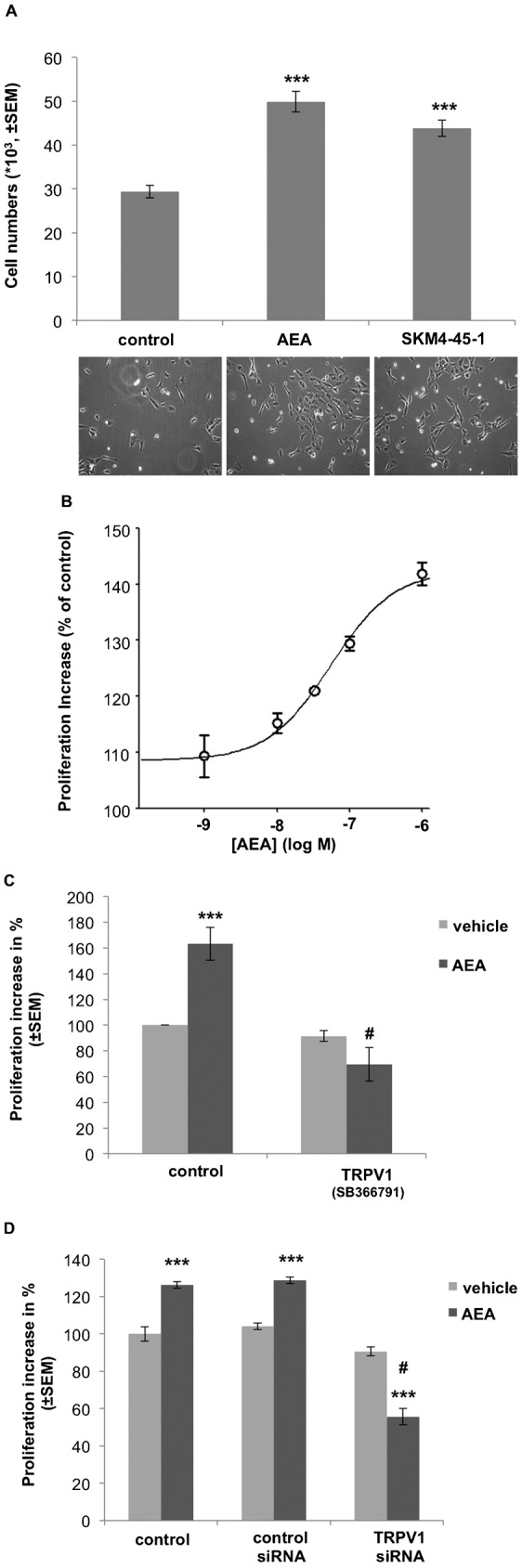
ECFC proliferation is promoted equally by SKM4-45-1 and AEA which is strongly reduced by inhibition of TRPV1. (A) Quantification of cell proliferation in response to anandamide (AEA) and SKM4-45-1. ECFCs where treated with or without 1 µM AEA (n = 10), 1 µM SKM4-45-1 (n = 3) (fluorescent AEA analogue) or same amounts vehicle. ECFC cell numbers were counted after 48 h treated with agonists. Values represent cell numbers (×10^3^; ± SEM); *** indicates p ≤ 0.001 significance as compared to vehicle control. Representative respective pictures of ECFC cell culture after 48 h treatment with vehicle (left picture), 1 µM AEA (middle picture) or 1 µM SKM4-45-1 (right picture) (at 10× magnification). (B) Concentration response curve of AEA on ECFC proliferation. ECFCs where treated with different concentrations of AEA (0.001; 0.01; 0.1 or 1 µM). Proliferation increase was compared to vehicle control (n = 3; ± SEM). (C) Columns represent cell proliferation increase. ECFCs were treated with vehicle (light grey bar) or anandamide (AEA, 1 µM; n = 10) (dark grey bar), with or without an antagonist against TRPV1 (SB366791, 10 µM; n = 3), as indicated. Effects are presented in % as compared to vehicle control (± SEM); *** indicates p ≤ 0.001 significance as compared to vehicle control. # indicates p ≤ 0.001 significance as compared to AEA treated cells without SB366791 treatment. (D) Columns represent cell proliferation increase of knock-down cells. ECFCs were treated with vehicle (light grey bar) or anandamide (AEA, 1 µM; n = 10) (dark grey bar), with or without transfection with non-specific siRNA or siRNA against TRPV1, as indicated. Effects are presented in % as compared to vehicle control (± SEM); *** indicates p ≤ 0.001 significance as compared to individual vehicle control. # indicates p ≤ 0.001 significance as compared to AEA treated cells without siRNA against TRPV1.

To explore whether or not the observed contribution of TRPV1 for AEA uptake is of physiological relevance, we investigated whether the observed TRPV1-mediated uptake of AEA is involved in the two major vascular functions of AEA, proliferation and network-formation. Therefore, ECFC proliferation and network-formation were tested and the contribution of TRPV1 was determined. Both, AEA (1 µM) as well as SKM4-45-1 (1 µM) were equipotent in promoting ECFC proliferation within 48 h by 69.7 ± 4.7% and 48.3 ± 4.3%, respectively (Fig. 5A). AEA stimulated ECFC proliferation in a concentration-dependent manner with an EC_50_ of 0.05 (0.04 to 0.08) µM (Fig. 5B). Notably, at a concentration of ≥10 µM AEA ECFCs did not proliferate within 48 h. To evaluate the involvement of TRPV1 during the AEA-induced (1 µM) proliferation increase, the TRPV1 inhibitor SB366791 (10 µM) was applied and proliferation. Within 48 h inhibition of TRPV1 counteracted the stimulatory effect of AEA (1 µM) on ECFC proliferation by 87.1 ± 13.2% (Fig. 5C). Genetic knock-down of TRPV1 in ECFC by a transient transfection with the respective siRNA showed a similar inhibitory effect on proliferation (Fig. 5D) and reversed the effect of AEA on ECFC proliferation (Fig. 5D). The TRPV1 agonist capsaicin (0.1 µM) increased ECFC proliferation by itself (app. 56.1 ± 5.6% of the effect of 1 µM AEA) while it prevented AEA-induced ECFC proliferation (supplementary material Fig. S4A).

**Fig. 6.**
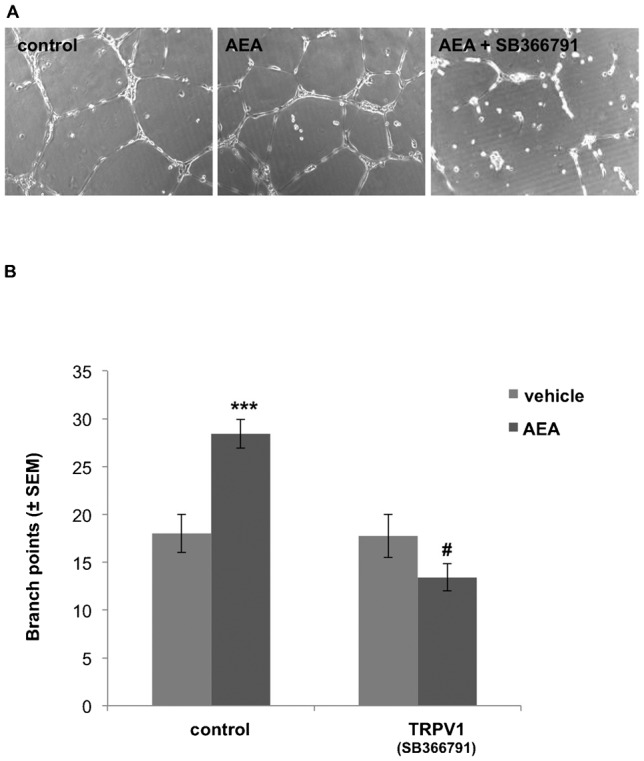
AEA-induced ECFC network-formation is efficiently reduced by the TRPV1 antagonist SB366791. (A) Representative pictures of ECFC network-formations after 12 h treatment with vehicle, anandamide (AEA, 1 µM; n = 10) or AEA + TRPV1-inhibitor (SB366791, 10 µM; n = 3), as depicted (at 10× magnification). (B) Columns represent number of branch-points formed by ECFCs. ECFCs were treated with vehicle (light grey bar) or anandamide (AEA, 1 µM; n = 10) (dark grey bar), with or without TRPV1 inhibitor (SB366791, 10 µM; n = 3), as indicated. Branch-point numbers were counted by ImageJ® (± SEM); *** indicates p ≤ 0.001 significance as compared to vehicle control. # indicates p ≤ 0.001 significance as compared to AEA only treatment.

Moreover, ECFC network-formation was tested on growth factor-reduced matrigel (Reinisch et al., 2009; Hofmann et al., 2012) under the influence of the TRPV1 antagonists SB366791. Within 12 h AEA (1 µM) significantly increased branch-point formation of ECFCs by 57.9 ± 1.5% as compared to the vehicle controls (Fig. 6A,B). Simultaneous treatment of ECFCs with AEA (1 µM) and the TRPV1 antagonist SB366791 (10 µM) prevented the stimulatory effect of AEA on ECFC network-formation (Fig. 6B), whereby SB366791 alone had no effect on network-formation.

## DISCUSSION

Moonlighting of a protein describes the performance of an additional, unrelated function apart from its established one. It has long been known that the endocannabinoid AEA activates the capsaicin receptor TRPV1 and leads to an increase of intracellular Ca^2+^-levels (Ralevic et al., 2001; van der Stelt et al., 2005; Waldeck-Weiermair et al., 2008; Ligresti et al., 2010). The herein presented study unmasks an unknown Ca^2+^-independent function of TRPV1 as contributor/regulator to/of AEA uptake into endothelial cells. We suggest that TRPV1-mediated AEA uptake is involved in the observed pro-angiogenic effect of AEA in endothelial cells. An active uptake mechanism would represent a bottleneck for the limitation of AEA function opening new possibilities for targeted pharmacotherapy against AEA-derived angiogenesis.

AEA was the first endocannabinoid to be discovered (Devane et al., 1992) and is associated with various pathological conditions including cardiovascular diseases requiring new vessel formation (e.g. pulmonary hypertension, cardiac ischemia/infarction) or tumor-angiogenesis. It is a matter of debate whether the observed pathological effects of AEA are dependent on its cellular uptake or relates to an extracellular binding. Interestingly, the freshly isolated ECFCs as well as the immortalized endothelial cell line EA.hy926 expressed the two classical endocannabinoid receptors CB_1_R and CB_2_R, the putative endocannabinoid orphan receptor GPR55 and TRPV1. The individual role of these receptors in AEA-mediated vascular function of ECFCs like cell proliferation and network-formation has not been investigated so far but might be of particular importance for the exploration of the therapeutic potential of the endocannabinoid system in regard of endothelial cell-vascular repair and neovascularization.

AEA has been shown to activate TRPV1 leading to an elevation of cytoplasmic Ca^2+^-levels (De Petrocellis et al., 2001; Ralevic et al., 2001; van der Stelt et al., 2005; Starowicz et al., 2007; Ligresti et al., 2010; Blumberg et al., 2011; Liao et al., 2013). Hence, it has been reported that depending on the integrin clustering status AEA-mediates different endothelial responses (Waldeck-Weiermair et al., 2008) while AEA failed to trigger cellular Ca^2+^ responses in the presence of extracellular Ca^2+^ in the EA.hy926 cells although this cell type expresses large amount of TRPV1 (Fig. 2A). In our present study we observed a concentration-dependent uptake of the AEA-analogue SKM4-45-1 in human endothelial colony-forming cells (ECFCs) and in the human umbilical vein endothelial cell-derived cell line EA.hy926 cells. Apparently, both AEA as well as SKM4-45-1 increase ECFC proliferation with a maximum pro-proliferative effect detected at 1 µM AEA, verifying the functional equality of SKM4-45-1 with AEA. Other studies (Muthian et al., 2000) have shown that AEA and SKM4-45-1 competed for the same binding site, suggesting the same uptake mechanism. Interestingly, the specific TRPV1 antagonist SB366791 (Gunthorpe et al., 2004) strongly reduced the cellular uptake of SKM4-45-1. Because we confirmed that SB366791 did not affect esterase activity, our data point to a new moonlighting function of TRPV1 during the AEA uptake in endothelial cells and their progenitors. Consistently, the TRPV1 agonist capsaicin (Caterina et al., 1997) reduced internalization of SKM4-45-1 in our experiments, thus, indicating that both substances compete for the same recently described (Cao et al., 2013; Liao et al., 2013) extracellular hydrophobic pocket of TRPV1. An additional explanation could be a desensitization of TRPV1 during capsaicin treatment leading to a reduced SKM4-45-1 uptake, however, the genuine mechanism must further be evaluated. Nevertheless, our data point to capsaicin as a potent inhibitor of AEA uptake, a phenomenon that might explain the inhibitory effect of capsaicin on tumor angiogenesis and growth (Min et al., 2004). Blocking of the classical cannabinoid receptors CB_1_R and CB_2_R or orphan receptor GPR55 with respective selective inhibitors (AM251 (Gatley et al., 1996; Pertwee et al., 2010), SR144528 (Rinaldi-Carmona et al., 1998) and CID16020046 (Kargl et al., 2013), respectively) had no significant effect on SKM4-45-1 uptake, thus, indicating that none of these receptors are involved in the uptake process of AEA in ECFCs.

Recently, by describing an inhibitory effect of the fatty acid amide hydrolase (FAAH) inhibitor methyl arachidonyl fluorophosphates (MAFP) (De Petrocellis et al., 1997; Deutsch et al., 1997; Martin et al., 2000; Glaser et al., 2003) on the AEA uptake of neuroblastoma and astrocytoma cells, the importance of FAAH activity for AEA uptake was demonstrated. However, in accordance to other reports (Högestätt et al., 2005; Costa et al., 2006), in the present work the FAAH inhibitor MAFP had no effect on SKM4-45-1 uptake, thus accumulation of SKM4-45-1 in ECFCs and EA.hy926 cells appears independent of FAAH activity. In contrast, cellular accumulation of SKM4-45-1 could be circumvented when applying the pan transport inhibitor AM404 that has been proposed to effect FAAH activity (Högestätt et al., 2005; Costa et al., 2006). However, AM404 was also described as TRPV1 agonist, like capsaicin (Högestätt et al., 2005). In view of this report and our presented findings that capsaicin prevented SKM4-45-1 uptake, we speculate that AM404 inhibits SKM4-45-1 uptake either by a competitive mechanism (AEA-like action) and/or as a TRPV1 agonist (as capsaicin-like action).

The crucial contribution of TRPV1 to AEA/SKM4-45-1 uptake into endothelial cells was further supported by our findings on the reduced SKM4-45-1 uptake in cells with diminuend TRPV1 expression while in cells overexpressing TRPV1 SKM4-45-1 uptake was boosted. Since removal of extracellular Ca^2+^ did not affect SKM4-45-1 uptake, the contribution of TRPV1 to AEA uptake appears independent from Ca^2+^. This is in contrast to our previous findings on the interplay between CB_1_R and GPR55 in the endothelial cell line EA.hy926 where removal of extracellular Ca^2+^ uncovers GPR55-dependent Ca^2+^ signaling upon AEA due to clustering of integrins that relieve the GPR55 from the inhibitory effect of the CB_1_R-signaling cascade (Waldeck-Weiermair et al., 2008). In addition to the interaction between two other AEA surface receptors, the present study reveals a moonlighting function of TRPV1 as contributor/regulator to/of AEA transport into endothelial cells.

The involvement of the endocannabinoid system during cancer formation and tumor-induced angiogenesis is jet unresolved (Maccarrone, 2013). Our present findings, along with the report of J. M. Rehman team (Opitz et al., 2007), who described that ECFCs release substantial amounts of AEA, allow us to hypothesize on a key function of AEA in the control of endothelial cells. At concentrations larger than 10 µM, AEA exhibited anti-proliferative and anti-angiogenic effects in human umbilical vein endothelial cells (HUVECs) and inhibited tumor-induced angiogenesis in a 3D tumor-angiogenesis model *in vitro* (Pisanti et al., 2007; Guindon and Hohmann, 2011). In accordance with these reports, in the present study, AEA at concentrations ≥10 µM failed to trigger ECFC proliferation and the cells subsequently detached. In contrast, it has been reported that AEA at concentrations ≤1 µM increases HUVECs proliferation and, via activation of CB_1_R initiates neovascularization in a mouse model of oxygen-induced retinopathy (Pisanti et al., 2011). Consistent with these reports, we see that low AEA concentrations (0.01–1 µM), resembling levels of endogenously produced endocannabinoids, also increased ECFC proliferation and network-formation. Hence, at least to some reports in the literature, the contradicting data arguing between a pro- (Pisanti et al., 2011) and anti-angiogenic (Pisanti et al., 2007) effect of AEA could in part result from cell specific differences in the expression levels/patterns of TRPV1 and the CBRs between HUVECs and ECFCs. Alternatively, our data suggest that the discrepancy to the previously described effects of AEA on angiogenesis could be a consequence of different AEA concentrations. In our studies we see a significant pro-proliferative and pro-angiogenic effect of AEA in concentrations ≤1 µM in ECFCs. These findings highlight the potential importance of the endocannabinoid system in activation of ECFCs to establish network/angiogenesis.

The data presented herein show that the effect of AEA on ECFC proliferation and network-formation essentially depends on TRPV1. Notably, the AEA-induced increase of ECFC proliferation correlated with active TRPV1 and its effect could be reversed in TRPV1 knock-down ECFCs. Further, inactivation of TRPV1 also prevented the AEA-induced branch point formation. In both assays ECFCs where strongly effected by inhibition of TRPV1 activity. Taken together our data strongly support the new paradigm that AEA might promote endothelial cell-established angiogenesis *in vivo* and therefore could participate in tumor-angiogenesis (Folkman, 1995; Pisanti et al., 2011).

Notably, elevated levels of AEA have been found in human glioblastomas and meningiomas (Petersen et al., 2005) and blocking of CB_1_R has been shown to inhibit tumor growth and angiogenesis (Pisanti et al., 2007; Pisanti et al., 2011). Accordingly, it is tempting to speculate that (certain) tumor cells might produce and release high levels of AEA to attract endothelial cells and subsequently lead to tumor-angiogenesis. Our present data suggest that the angiogenic effect of tumor-derived AEA on endothelial cells essentially involves TRPV1-mediated uptake of AEA, subsequently followed by activation of intracellular AEA receptors (Rozenfeld and Devi, 2008; Brailoiu et al., 2011; den Boon et al., 2012; Fowler, 2013) yielding the pro-angiogenic effect of this endocannabinoid.

Our present data may imply a novel approach to circumvent AEA-triggered tumor-angiogenesis of endothelial cells by specifically targeting the putative AEA transport-regulator TRPV1. In contrast, our data suggest that AEA application could promote neo-vascularization in cases of tissue ischemia as after heart attack or metabolic syndrome though such a neo-vascularization therapy might imply the risk to support tumor growth. Though exceeding the scope of the present study, the contribution of moonlighting TRPV1 in AEA-stimulated tumor-angiogenesis by endothelial cells awaits further investigation in order to evaluate the therapeutic potential of TRPV1 inhibition against tumor-angiogenesis.

## MATERIALS AND METHODS

### Cell culture and characterization

ECFCs were isolated from neonatal cord and their distinct endothelial phenotype verified by flow-cytometry (supplementary material Fig. S4B) as previously described (Reinisch et al., 2009; Hofmann et al., 2012). Three different ECFC donors were grown in endothelial growth medium-2 (EGM-2) (Lonza; Basel, Switzerland) containing 2% FBS. The human umbilical vein derived endothelial cell line EA.hy926 was grown in DMEM containing 10% FBS and 1% hypoxanthine-aminopterin-thymidine selection medium (HAT) (PAA Laboratories, Pasching, Austria). The cell lines Hela and human embryonic kidney cells (Hek293) were grown in DMEM containing 10% FBS.

### Reduction of TRPV1 gene expression by siRNA

Transfection of EA.hy926 cells with a pool of 3 target-specific TRPV1 siRNA (VR1 siRNA; sc36826; Santa Cruz, CA, USA), was performed using Transfast^TM^ transfection reagent (Promega, Madison, WI, USA) according to the manufacturer's protocol. All experiments were performed 48 hours after transfection. The efficiency of siRNAs and of an appropriate negative control was approved by real-time, quantitative PCR and western blot.

### Overexpression of red fluorescence protein (RFP)-TRPV1

A recombinant (RFP)-TRPV1 was constructed in our laboratory. Briefly, cloning was performed according to standard procedures and all products were verified by sequencing. The plasmid pENTR201, which contains the TRPV1 coding sequence NM_080704.3 (ORFeome Collaboration Human TRPV1 ORF (Thermo Fisher, Waltham, MA, USA)) was used as template in a PCR with following primers: TRPV1_for 5′-GGATCGATATGAAGAAATGGAGCAGCAC-3′, which adds a ClaI restriction site and TRPV1_rev 5′-AAAAGCTTTCACTTCTCCCCGGAAGCGG-3′, which adds a Stopcodon and a HindIII restriction site. To obtain an N-terminal tagged TRPV1, the PCR product was subcloned into a RFP containing pcDNA3.1^−^ vector. EA.hy926 cells were transfected with protein constructs as described earlier. The efficiency of overexpression and of an appropriate negative control was approved by real-time, quantitative PCR and western blot.

### Isolation of total RNA and cDNA synthesis

Total cellular RNA was extracted using the PeqGOLD Total RNA Kit (PEQLAB Biotechnologie GmbH, Erlangen, Germany) and quantified spectrophotometrically at 260/280 nm. For cDNA preparation, 2 µg of total RNA were reverse transcribed using the High-Capacity cDNA Reverse Transcription Kit (Life Technologies, Gibco, Invitrogen, Vienna, Austria) including 1 µl RNasin® Plus RNAse Inhibitor (Promega) following the manufacturer's instructions.

### Conventional RT-PCR

Conventional PCR was carried out in a personal Thermocycler (Primus 25 advanced, PEQLAB) using GoTaq® G2 Hot Start Green Master Mix (Promega, no. M7423) following the manufacturer's instructions. PCR profiles were as follows: an initial denaturation step at 95°C for 2.5 min, followed by 27 cycles (β-actin) or 30 cycles (TRPV1) with 1 min denaturation (95°C), 30 s annealing (60°C for β-actin; 58°C for TRPV1) and 30 s elongation (72°C), and a final extension of 7 min (72°C). PCR products (20 µl) were separated by standard gel electrophoresis on 1.5% agarose gels at 70 V for 60 min in 1× TAE buffer containing 5 µl/ml Roti®-GelStain (Carl ROTH GmbH, no. 3865.1). Digitized UV-images of the gels were taken with a gel documentation system (GenoSmart, VWR). PCR-primers were chosen to span at least one exon/intron boundary to minimize amplification of contaminating genomic DNA. Primer sequences were as follows: TRPV1, forward 5′-CAGCAGCGAGACCCCTAATC-3′ and reverse 5′-GCTGTCCACAAACAGGGTCT-3′ (amplicon 322 bp); CB1, forward 5′-TCCTACCACTTCATCGGCAG-3′ and reverse 5′-CACGGCGATCACAATGGCTA-3′ (amplicon 294 bp); CB2, forward 5′-CCGCCATTGACCGATACCTC-3′ and reverse 5′-TGGCCAACCTCACATCCAGC-3′ (amplicon 354 bp); GPR55, forward 5′-GCTGCCACCTCCATCTACAT-3′ and reverse 5′-CGCTCCAGGTATCATCAGAC-3′ (amplicon 367 bp); and β-Actin (as housekeeping gene), forward 5′-GCAAGAGAGTCCTCACC-3′ and reverse 5′-GCACAGCCTGGATAGCAACG-3′ (amplicon 240 bp). Band intensities were compared by ImageJ (National Institutes of Health, Bethesda, MD).

### Quantitative RealTime-PCR (qRT-PCR)

The efficiency of siRNA knock-down as well as TRPV1 over-expression was quantified by real-time PCR. qRT-PCR was carried out in a Light Cycler 480 System (Roche, Basel, Switzerland) using the GoTaq® qPCR Master Mix (Promega; no. A6002) following the manufacturer's instructions. Gene-specific primers used for qRT-PCR were as follows: TRPV1, forward 5′-GACCACCTGGAACACCAACG-3′ and reverse 5′-TGAGCAGACTGCCTATCTCG-3′ (amplicon 177 bp), and QuantiTect® Primer Assay (Qiagen, Hilden, Germany) for human GAPDH (no. QT01192646) as housekeeping gene. Real-time amplification data were analyzed using the REST-MCS beta software version 2 [August 2006]. PCR reactions were examined visually for primer-dimers and other non-specific amplifications on 2% agarose gels.

### Western blot analysis

Western blots were performed according to standard protocols. Cells were directly lyzed with 1× Laemmli sample buffer, heat denatured and resolved in a 4–20% SDS-PAGE (Bio-Rad) in parallel to full-range rainbow^TM^ molecular weight marker (GE Healthcare, Munich, Germany) and transferred onto a PVDF-membrane (Millipore). Specific proteins were detected using antibodies against CB_1_R (Abcam), CB_2_R (Thermo Scientific), GPR55 (Thermo Scientific) and TRPV1 (Cell Signaling) antibodies compared to house-keeping protein control β-actin (Santa Cruz), followed by anti-mouse (Cell Signaling) or anti-rabbit (Abcam) HRP antibody and visualized with ‘super signal west pico luminol/enhancer developing solution’ (Thermo Scientific).

### Anandamide uptake studies

Three different ECFC donors and EA.hy926 cells were grown to 70% confluence in either 8-well Ibidi treated slides (Ibidi, Planegg, Germany) or on glass cover slips (Ø  =  30 mm), placed in a 6-well plate (Nunc). The fluorescent AEA analogue SKM-4-45-1 (Cayman Chemical Europe, Tallinn, Estonia) was applied to ECFCs and EA.hy926 in concentrations as indicated in conventional or Ca^2+^-free EGTA buffered medium. Fluorescence increase was documented with a Zeiss LSM 410 microscope (Zeiss, Jena, Germany) on 10× magnification by obtaining serial pictures every 10 seconds throughout 30 minutes. Single cell fluorescence increase was analyzed by VisiView® (Visitron Systems, Puchheim, Germany). Changes in SKM4-45-1 uptake was detected similarly in ECFCs and EA.hy926 in the presence of anandamide (0.001–30 µM), CID16020046 (20 µM), SB366791 (10 µM) and Capsaicin (0.1 µM) (all obtained by Tocris Bioscience, Bristol, UK), AM404 (10 µM), AM251 (0.1 µM), SR144528 (1 µM), Methyl Arachidonyl Fluorophosphonate (MAFP, 0.1 µM) (all obtained from Cayman Chemical Europe, Tallinn, Estonia) as well as after treatment with siRNA against TRPV1 and RFP-TRPV1. Changes in Fluo-4/AM (Life Technologies) fluorescence intensity were observed as described with and without SB366791 pre-treatment. High resolution imaging of 100× magnification of TRPV1 overexpressing EA.hy926 cells was performed using the same confocal microscope.

### Proliferation assay

Three different ECFC donors were seeded in 6-well plates (Nalge Nunc, Rochester, NY) in EGM-2 at a density of 3,000 c/cm^2^ and allowed to adhere for 24 hours. Subsequently, cells were subjected to growth factor reduced medium with or without AEA and different endocannabinoid receptor agonists and antagonists as indicated. The substances anandamide, CID16020046, SB366791 and Capsaicin were obtained by Tocris Bioscience (Northpoint, Avonmouth, Bristol, UK). SKM4-45-1, AM251, SR144528 (Cayman Chemical Europe, Tallinn, Estonia). After 48 hours treatment cells were harvested and cell numbers analyzed by a Casy cell counter (Roche, Mannheim, Germany).

### *In vitro* angiogenesis assay

Capillary-like network formation of three different ECFC donors on growth factor reduced Matrigel® (BD, Biosciences, San Jose, CA, USA) was performed according to instruction manual. Influence of different endocannabinoid receptor agonists and antagonists were tested as indicated. Network formation (12–14 hours) was documented with a Nikon Coolpix 4500 camera on a Nikon TMS-F microscope (Nikon, Amsterdam, Netherlands). Branch-points were counted by ImageJ (National Institutes of Health, Bethesda, MD).

### Statistics

All experiments were performed with three donors of ECFCs and the EA.hy926 cells in at least triplicate. n values refer to number of experimental days. Data were compared using ANOVA and subsequent Bonferroni *post hoc* test or two-tailed Student's t-test assuming unequal variances. Statistical significance was assumed when p<0.05. EC50 values are given as the mean and the 95% confidential interval in parenthesis.

### List of abbreviations

AEA, anandamide; CBRs, cannabinoid receptors; TRPV1, transient receptor potential vanilloid 1; EA.hy926, human endothelial vein cell line; ECFCs, endothelial colony-forming cells; FAAH, fatty acid amide hydrolase; GPR, G-protein coupled receptors; MAFP, methyl arachidonyl fluorophosphonate.

## Supplementary Material

Supplementary Material
