# Supplementary material for: TRPV1 mediates cellular uptake of anandamide and thus promotes endothelial cell proliferation and network-formation
Source: Biol Open. 2014 Nov 13;3(12):1164–72. doi: 10.1242/bio.20149571 (PMC4265754; doi:10.1242/bio.20149571)
Supplement: Supplementary Material [file supp_3_12_1164__index.html]

TRPV1 mediates cellular uptake of anandamide and thus promotes endothelial cell proliferation and network-formation — TRPV1 mediates cellular uptake of anandamide and thus promotes endothelial cell proliferation and network-formation — Supplementary Material 

# TRPV1 mediates cellular uptake of anandamide and thus promotes endothelial cell proliferation and network-formation

## bio.20149571 Supplementary Material

**Files in this Data Supplement:**

- Supplementary Material - Nicole A. Hofmann et al. doi: 10.1242/bio.20149571
