# Supplementary material for: TRPV1 mediates cellular uptake of anandamide and thus promotes endothelial cell proliferation and network-formation
Source: Biol Open. 2014 Nov 13;3(12):1164–72. doi: 10.1242/bio.20149571 (PMC4265754; doi:10.1242/bio.20149571)
Supplement: Supplementary Material [file supp_bio.20149571_bio.20149571-s1.pdf]

Biology Open (2014) 000, 1–9 doi:10.1242/bio.20149571

Supplementary Material

Nicole A. Hofmann et al. doi: 10.1242/bio.20149571

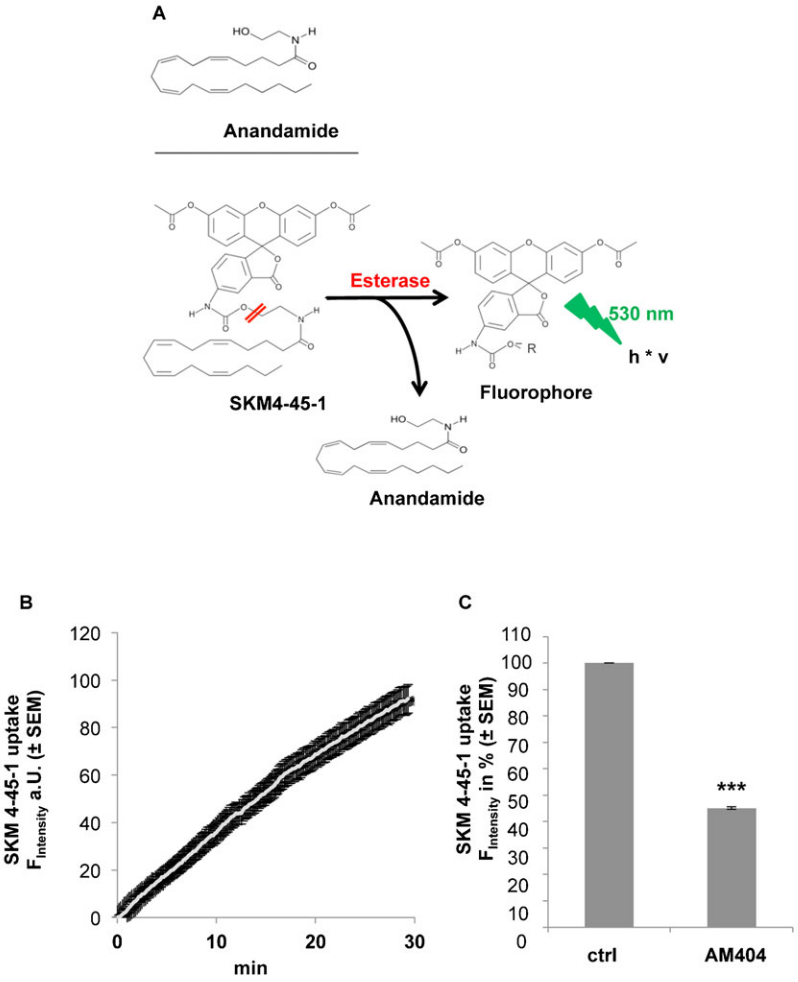

Fig. S1. (A) Structure of AEA and its analog SKM4-45-1 and scheme how uptake of SKM4-45-1 yields fluorescence upon cleavage. Scheme shows the structural formula of AEA and SKM4-45-4. Lower panel shows the intracellular cleavage of SKM4-45-1 by esterases resulting in release of anandamide and a fluorophore emitting light by 530 nm. (B) Accumulation of SKM4-45-1 (1 μM) was detected by confocal microscopy (530 nm) by measuring increase in fluorescence (arbitrary units, a.U.) throughout 30 minutes (n=8; ± SEM). (C) ECFC SKM4-45-1 accumulation can be blocked by the transport inhibitor AM404. Accumulation of SKM4-45-1 (1 μM) in ECFCs with or without AM404 (10 μM) was detected by confocal microscopy after 30 minutes and is shown in % versus SKM4-45-1 uptake without AM404 (n=5; ± SEM). *** reflects $p \leq 0.001$ significance as compared to vehicle control.

Biology Open

Biology Open (2014) 000, 1–9 doi:10.1242/bio.20149571

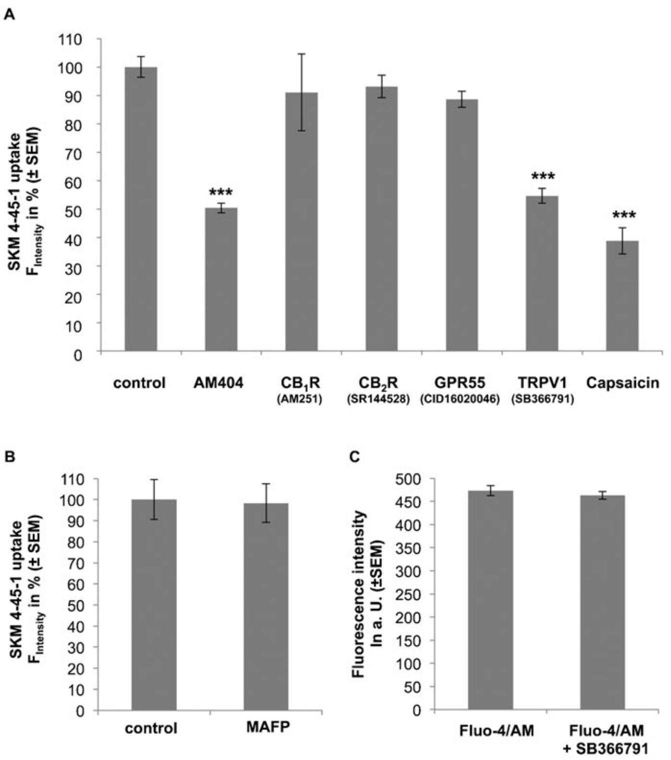

Fig. S2. (A) Pharmacological inhibition of TRPV1 in EA.hy926 inhibits AEA uptake. SKM 4-45-1 (1 µM) was added to EA.hy926s with or without treatment with antagonists against pantransporter (AM404; 10 µM; n=5), CB1R (AM251, 0.1 µM; n=4), CB2R (SR144528, 1 µM; n=3), GPR55 (CID16020046, 20 µM; n=8) and TRPV1 (SB366791, 10 µM; n=3). Increase in intracellular fluorescence intensity (530 nm) was measured after 30 minutes by confocal microscopy. Values are shown as % fluorescence compared to vehicle control (± SEM). *** reflects $p \leq 0.001$ significance as compared to vehicle control. (B,C) The fatty acid amide hydrolase (FAAH) inhibitor methyl arachidonyl fluorophonate (MAFP) does not affect uptake of SKM4-45-1 in ECFCs. Inhibition of TRPV1 by its selective inhibitor SB366791 had no effect on the cellular esterase activity. (B) Inhibition of fatty acid amide hydrolase (FAAH) does not influence SKM4-45-1 uptake. SKM4-45-1 (1 µM; n=4) was added to ECFCs with vehicle or antagonists against FAAH methyl arachidonyl fluorophosphonate (MAFP) (0.1 µM; n=4). Increase in intracellular fluorescence intensity (530 nm) was measured after 30 minutes by confocal microscopy. Values are shown as % fluorescence as compared to vehicle control (± SEM). (C) Fluo-2/AM (1 µM) was added to ECFCs with vehicle or SB366791 (10 µM). Esterase dependent intracellular fluorescence (530 nm) was measured after 30 minutes by confocal microscopy. Values are shown as % fluorescence as compared to controls without inhibitors (n=3; ± SEM).

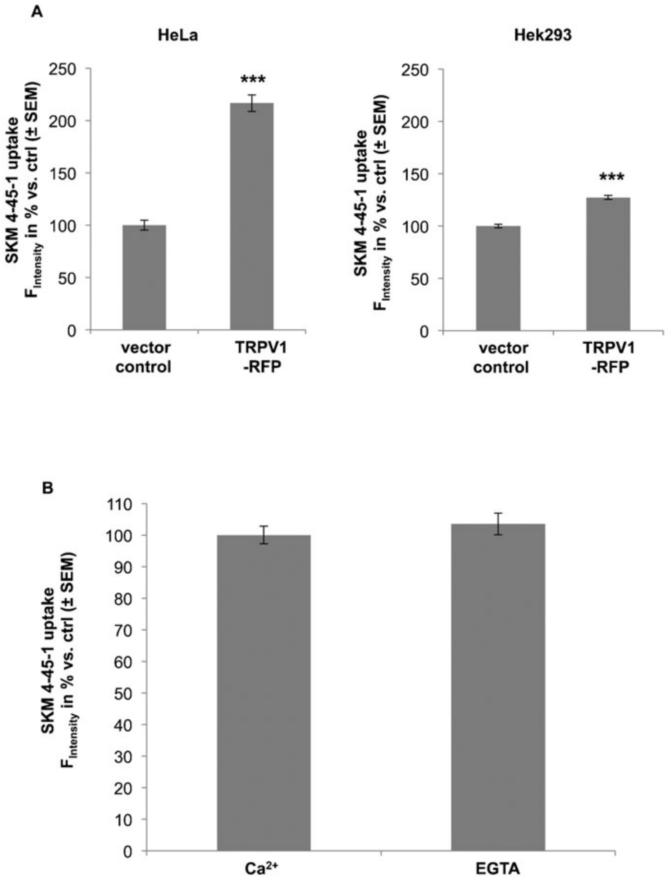

Fig. S3. (A) Columns represent SKM4-45-1 accumulation in Hela and human embryonic kidney cells (Hek293) with vector control or TRPV1-RFP transfection. SKM4-45-1 uptake was measured after 30 minutes incubation. Values show % fluorescence as compared to vector controls (n=3; ± SEM); *** reflects $p \leq 0.001$ significance as compared to vector control cells. (B) SKM4-45-1 uptake is independent of the presence of extracellular Ca^{2+}. SKM 4-45-1 (1 µM) was added to ECFC with conventional Ca^{2+}-containing or Ca^{2+}-free (i.e. EGTA containing) medium. Increase in intracellular fluorescence intensity (530 nm) was measured after 30 minutes by confocal microscopy. Values are shown as % fluorescence compared to vehicle controls (n=4; ± SEM).

Biology Open (2014) 000, 1–9 doi:10.1242/bio.20149571

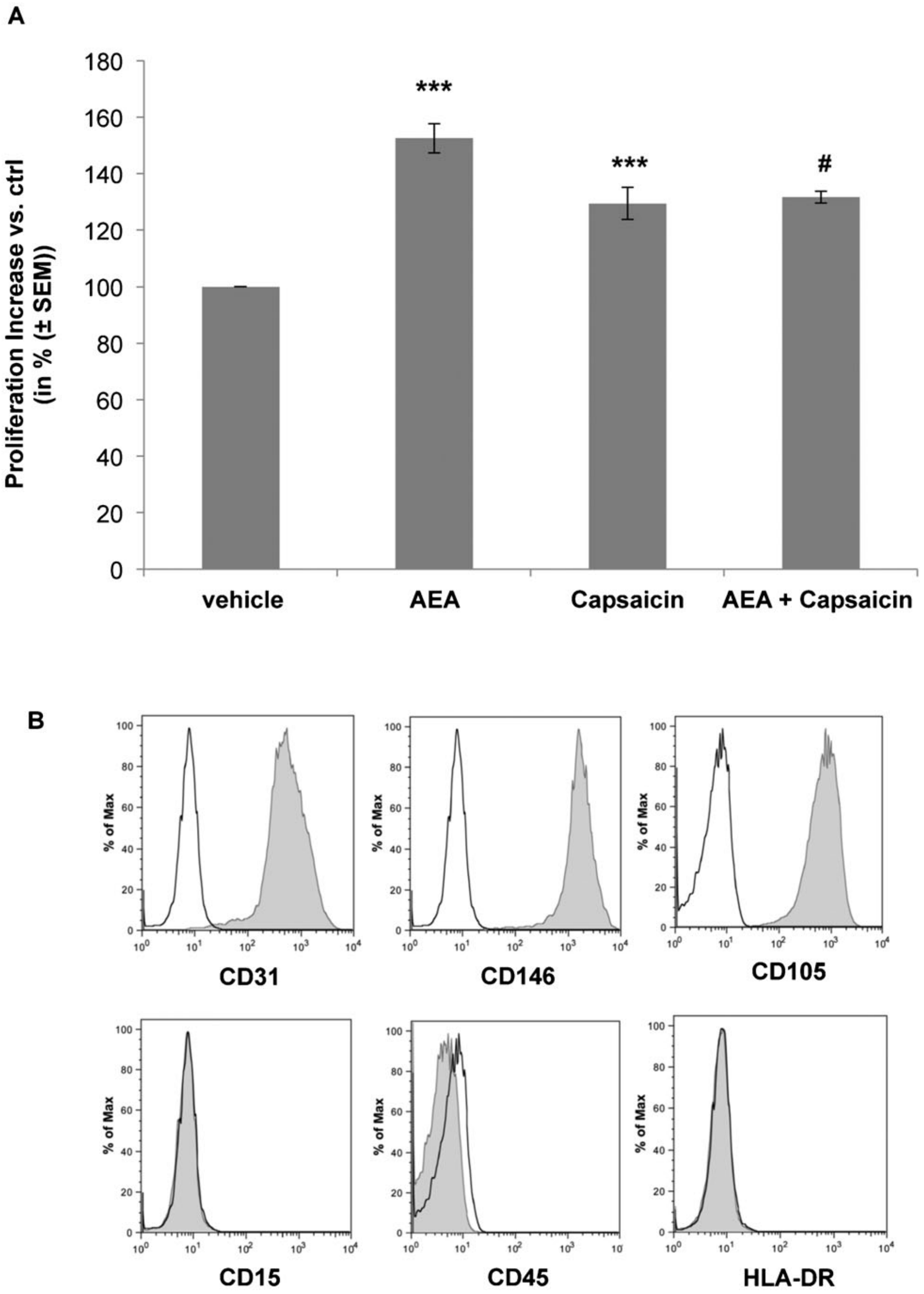

Fig. S4. (A) The TRPV1 agonist capsaicin prevents AEA-induced proliferation of ECFCs. ECFCs where treated with vehicle or either 1 μM AEA (n=10), 0.1 μM capsaicin (TRPV1 agonist; n=4) or both. Values are shown as % fluorescence as compared to vehicle control (± SEM). *** reflects $p \leq 0.001$ significance as compared to vehicle control. # reflects $p \leq 0.05$ significance as compared AEA treatment only. (B) Phenotypical characterization of ECFCs. Representative flow cytometry histograms of ECFCs showing reactivity with EC-expressed molecules (right-shifted filled gray curves compared with black lined open curves of the appropriate isotype controls) and lack of reactivity with hematopoietic (CD15 and CD45) and activation (human leukocyte antigen class II type DR, HLA-DR) markers.

Biology Open
